# Can the Energy and Enterprise Initiative Improve Public Health?

**DOI:** 10.1289/ehp.120-a347

**Published:** 2012-08-31

**Authors:** David C. Holzman

**Affiliations:** David C. Holzman writes on science, medicine, energy, economics, and cars from Lexington and Wellfleet, MA. His work has appeared in *Smithsonian*, *The Atlantic Monthly*, and the *Journal of the National Cancer Institute*.

A former Republican congressman from South Carolina has started an institution to promote conservative approaches to mitigating climate change and achieving energy security for the United States. According to Bob Inglis, whose Energy and Enterprise Initiative is housed within George Mason University, market distortions lead Americans to use far more fossil fuels than they would if they paid the full cost of that fuel. If fuel producers are held fully accountable for the health, productivity, and environmental costs of their products, he says, free enterprise will naturally give rise to the cleanest, most sustainable fuels.

A committee of the National Research Council recently estimated that burning fossil fuels for electricity, heat, and vehicle operation cost the United States more than $120 billion in 2005. These estimates were based on damages to human health, grain crop and timber yields, building materials, and recreation by criteria air pollutants including particulate matter, sulfur dioxide, and nitrogen oxides.^1^

The majority of this cost was attributed to premature deaths resulting from exposure to these pollutants. The estimates omitted a number of factors that could further increase costs, including damages to ecosystem services, environmental damages from coal mining, and military costs of protecting fuel imports.^1^

Alexander Bozmoski, director of strategy and operations for the Energy and Enterprise Initiative, says society bears those costs not through payments at the gasoline pump or on their power bill, but instead through greater health insurance premiums, lost income, and increased taxes for defense spending.

In 2009 the United States provided $5.3 billion in subsidies for fossil fuel producers and $4.9 billion in subsidies for fossil fuel consumers. Of the total $10.2 billion in producer and consumer subsidies, more than half went toward natural gas, one-third went toward petroleum, and about one-tenth went toward coal. These figures do not include costs for fossil energy research and development or for maintenance of the Strategic Petroleum Reserve or the Northeast Home Heating Oil Reserve, which were established as buffers against major supply disruptions.^2^

**Hidden Costs of Fossil Fuels**

According to the National Research Council report *Hidden Costs of Energy: Unpriced Consequences of Energy Production and Use*,^1^ criteria air pollutants emitted by power plants and motor vehicles caused more than $120 billion in estimated external costs in 2005:

- $62 billion in damage was attributed to 406 coal-fired electric power plants. Damages averaged $156 million per plant and 3.2¢ per kilowatt-hour of electricity produced.
- $740 million in damage was attributed to 498 natural gas electric power plants. Damages averaged $1.49 million per plant and 0.16¢ per kilowatt-hour of electricity produced.
- $1.4 billion in damage was attributed to burning natural gas for heating (residential, commercial, and industrial). Median estimated damages were about 11¢ per thousand cubic feet of heated space.
- $56 billion in damage was attributed to motor vehicles. Damages averaged 1.2–1.7¢ per mile traveled depending on vehicle technology and fuel type.

Fossil fuel subsidies come in a variety of forms. Some of the largest subsidies on the production side include write-offs for properties used to process qualified petroleum fuels and costs associated with exploration and development for oil and gas wells. Consumer subsidies include the Low-Income Energy Assistance Program to help needy residents pay their energy bills, fuel tax exemptions for farmers (one of the biggest single subsidies at nearly $1 billion in 2009), and a tax credit for investing in power generation projects that use “clean coal” technologies.^2^

The United States is not alone in subsidizing these fuels; worldwide, fossil fuel subsidies amounted to $409 billion in 2010.^3^ In a 2012 interview with the U.K. *Guardian*, Fatih Birol, the chief economist at the International Energy Agency, was quoted as saying, “Energy is significantly underpriced in many parts of the world, leading to wasteful consumption, price volatility, and fuel smuggling. It’s also undermining the competitiveness of renewables.” ^4^

According to Birol, phasing out subsidies could, in the course of discouraging waste and encouraging development of clean fuels, reduce emissions by about half the amount necessary to prevent warming beyond 2°C over preindustrial levels.^4^ That is the goal many governments worldwide have adopted to avoid what are projected to be the most serious effects of climate change.^5^

Inglis says the initiative’s primary modus operandi will be educational: to provide information to conservatives, particularly young conservatives, by speaking at colleges and universities, convening forums, and sponsoring policy papers. The initiative website is clear that its founders “don’t subscribe to apocalyptic visions of climate change.” They do believe, however, that “the best science available indicates that America faces substantial risks in a changing climate [and] that conservative energy policy should mitigate those risks.”^6^
